# Uncertainty in weather and climate prediction

**DOI:** 10.1098/rsta.2011.0161

**Published:** 2011-12-13

**Authors:** Julia Slingo, Tim Palmer

**Affiliations:** 1 Met Office, Exeter, UK; 2 European Centre for Medium-Range Weather Forecasting, , Reading, Berkshire, UK; 3 Department of Physics, University of Oxford, Oxford, UK

**Keywords:** uncertainty, climate prediction, weather forecasting, probabilities, ensemble prediction system

## Abstract

Following Lorenz's seminal work on chaos theory in the 1960s, probabilistic approaches to prediction have come to dominate the science of weather and climate forecasting. This paper gives a perspective on Lorenz's work and how it has influenced the ways in which we seek to represent uncertainty in forecasts on all lead times from hours to decades. It looks at how model uncertainty has been represented in probabilistic prediction systems and considers the challenges posed by a changing climate. Finally, the paper considers how the uncertainty in projections of climate change can be addressed to deliver more reliable and confident assessments that support decision-making on adaptation and mitigation.

… one flap of a sea-gull's wing may forever change the future course of the weather.

Lorenz [1, p. 19]

## 1. Introduction

In 1963, Lorenz published his seminal paper on ‘Deterministic non-periodic flow’, which was to change the course of weather and climate prediction profoundly over the following decades and to embed the theory of chaos at the heart of meteorology. Indeed, it could be said that his view of the atmosphere (and subsequently also the oceans) as a chaotic system has coloured our thinking of the predictability of weather and subsequently climate from thereon.

**Figure 1.**
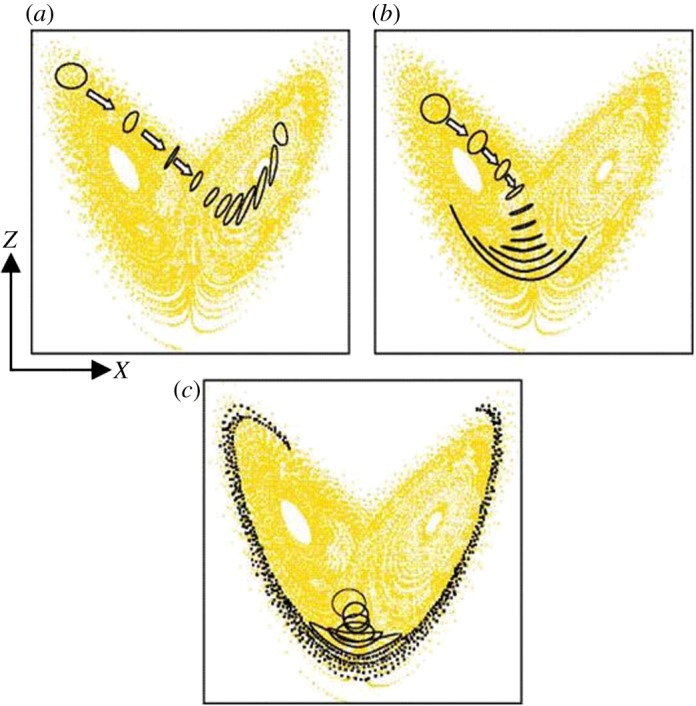
Examples of finite-time error growth on the Lorenz attractor for three probabilistic predictions starting from different points on the attractor. (*a*) High predictability and therefore a high level of confidence in the transition to a different ‘weather’ regime. (*b*) A high level of predictability in the near term but then increasing uncertainty later in the forecast with a modest probability of a transition to a different ‘weather’ regime. (*c*) A forecast starting near the transition point between regimes is highly uncertain.

Lorenz was able to show that even for a simple set of nonlinear equations (1.1), the evolution of the solution could be changed by minute perturbations to the initial conditions, in other words, beyond a certain forecast lead time, there is no longer a single, deterministic solution and hence all forecasts must be treated as probabilistic. The fractionally dimensioned space occupied by the trajectories of the solutions of these nonlinear equations became known as the Lorenz attractor (figure 1), which suggests that nonlinear systems, such as the atmosphere, may exhibit regime-like structures that are, although fully deterministic, subject to abrupt and seemingly random change.
1.1
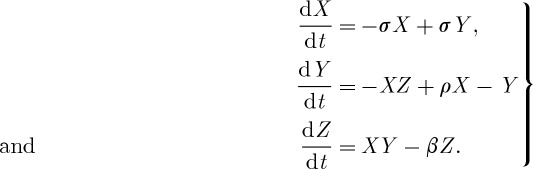

But more importantly, the Lorenz [2] model also indicates that the predictability of a chaotic system is flow dependent, so that while some weather patterns or regimes may be highly unpredictable, others may contain substantial predictability; in other words, the predictability is itself both variable and predictable (figure 1). This property has fundamental implications for weather and climate prediction as it allows an assessment of the reliability and hence confidence in the probability distribution of the forecasts.

In a later, but highly prescient paper, Lorenz [1] also considered the interplay of various scales of motion in determining the predictability of a system. The results showed that errors at the cumulus scale can invade the errors at the synoptic scale in two days and infect the very largest scales in two weeks. Thirty years later, the relevance of this study has been realized in the development of stochastic approaches to represent cumulus convection and its upscale energy transports, and in the emerging efforts to resolve these multi-scale processes in atmospheric simulations at the cloud system-resolving scale (approx. 1 km).

This paper considers how chaos theory has shaped our approach to numerical weather prediction, why, despite the limits to atmospheric predictability suggested by Lorenz, seasonal and even decadal prediction is possible, and how uncertainty should be addressed in the context of climate change. Finally, some recommendations for future progress towards more confident and reliable predictions in the face of uncertainty are considered.

## 2. Handling uncertainty in weather forecasting

Lorenz showed clearly that the uncertainty in the initial condition, however small, will lead to uncertainty in the forecast after a certain, but variable, period of time depending on the initial state of the atmosphere. As a consequence, the numerical weather prediction community began to consider the use of probabilistic methods for forecasting, especially beyond the deterministic limit of one week or so suggested by Lorenz.

**Figure 2.**
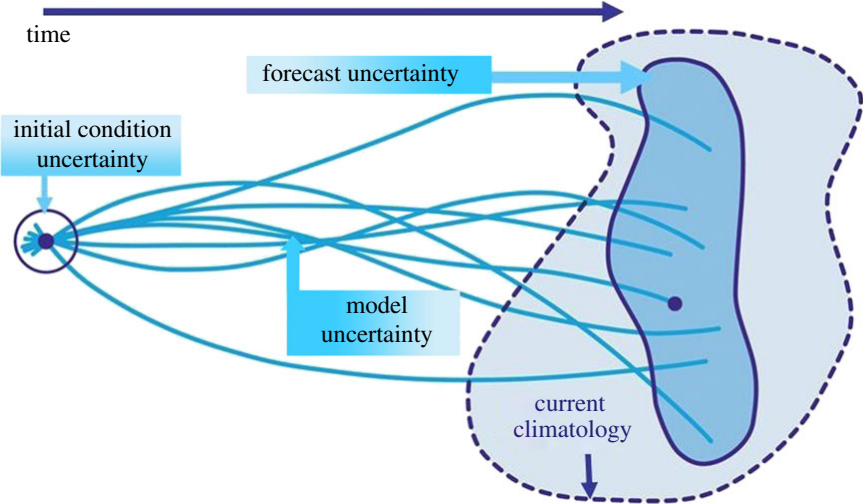
Schematic of a probabilistic weather forecast using initial condition uncertainties. The blue lines show the trajectories of the individual forecasts that diverge from each other owing to uncertainties in the initial conditions and in the representation of sub-gridscale processes in the model. The dashed, lighter blue envelope represents the range of possible states that the real atmosphere could encompass and the solid, dark blue envelope represents the range of states sampled by the model predictions.

Early implementation of probabilistic methods for numerical weather prediction was based on applying small, random perturbations to the atmospheric state variables (temperature, humidity, winds and pressure) in the analysed initial condition. Because the atmosphere is nonlinear, these minute perturbations are then amplified by chaotic processes and each forecast diverges from the others (figure 2).

**Figure 3.**
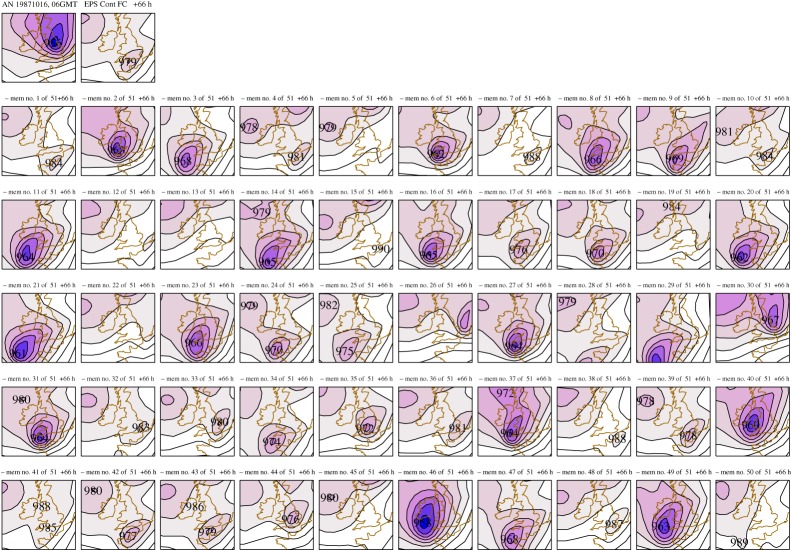
Example of 66 h probabilistic forecast for 15–16 October 1987. Top left shows the analysed deep depression with damaging winds on its southern flank. Top right shows the deterministic forecast, and the remaining 50 panels show other possible outcomes based on perturbations to the initial conditions. A substantial fraction of the ensemble indicates the development of a deep depression.

The advantages of ensemble forecasting became readily apparent in the case of the October 1987 storm when Michael Fish, a British Broadcasting Corporation (BBC) weather forecaster, was famously quoted as saying ‘…….*a woman rang the BBC and said she had heard that there was a hurricane on the way. Well if you are watching, don't worry there isn't*’. He had access to only a single deterministic forecast, which gave no clues as to what might happen. If he had had access to a fully probabilistic system, then he might well have decided to issue a warning of severe weather (figure 3).

Since then, probabilistic weather forecasting has become routine and its advantages are now widely appreciated. It provides a range of plausible forecast solutions, which allows the forecaster to assess possible outcomes, estimate the risks and probabilities of those outcomes and to gauge the level of confidence in the final forecast. From the users' perspective, the forecast probabilities allow them to decide on the level of risk they are prepared to take depending on their vulnerabilities, and to take appropriate action within a proper understanding of the uncertainties.

**Figure 4.**
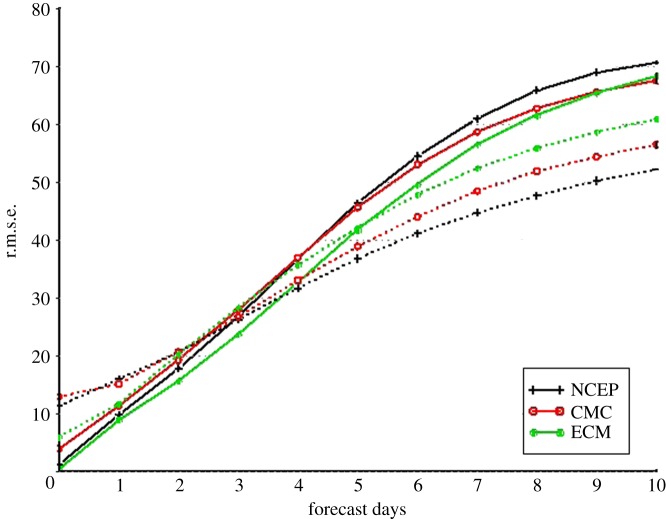
Statistics of ensemble mean forecast error (r.m.s.e.; solid line) and ensemble spread (dotted line) in Northern Hemisphere from three ensemble prediction systems (NCEP, National Center for Environmental Prediction; CMC, Meteorological Service of Canada; ECM, European Centre for Medium-range Weather Forecasting). Note that the forecast error is based on an anomaly forecast and therefore does not include the model systematic bias.

However, the effectiveness of probabilistic methods in weather forecasting depends on the reliability of the ensemble, where reliability in this context means that there is a proper representation of the forecast uncertainties. It became clear fairly early on that the systems based just on random perturbations to the initial conditions were not sufficiently dispersive and that the range of solutions did not fill the phase space of possible states for the real atmosphere ([3]; figure 4). The root mean square error of the ensemble mean anomaly forecast grows faster than the spread, which indicates that the ensemble is under-dispersive and hence the ensemble forecast is over-confident.

This lack of spread suggested that the perturbations to the initial conditions were sub-optimal and did not necessarily capture the most rapidly growing modes [4]. Considerable research into identifying those modes using singular vector techniques was undertaken [5], and significant progress achieved in increasing the spread of the ensemble. Nevertheless, the ensemble remained under-dispersive so something else must be missing.

Numerous atmospheric model studies and intercomparisons [6] have shown that there is a large divergence in the quality of simulations that can be related to model formulation, especially the parametrization of physical processes related to diabatic heating (e.g. cumulus convection, cloud–radiation interactions and boundary-layer turbulent fluxes). Although these parametrizations are based on fundamental physics, empirical assumptions have to be made in order to represent what are essentially sub-gridscale processes at the resolved scale of the model. The usual approach is to define what are called bulk formulae, which seek to represent empirically the statistical properties of the sub-gridscale processes and how they relate to the large-scale, resolved state of the atmosphere (or ocean). Traditionally, a deterministic approach has been taken to define these bulk formulae and their closure parameters, despite the fact that considerable uncertainty exists and observations suggest a significant range of possible formulations and values.

Therefore, it is clear that uncertainty in the model itself, and systematic biases in the model's simulation lead to restricted sampling of the forecast phase space and under-dispersion in the ensemble. Early studies investigated the role of model uncertainty and used random perturbations to the increments from the physical parametrizations, time step by time step [7], while other approaches used random variations to the closure parameters in the physical parametrizations. These were based on a range of observational estimates and expert judgement, and were kept fixed throughout the particular simulation—the perturbed parameter approach. Both led to some improvements in the ensemble spread, but as will be discussed later, the perturbed parameter approach has proved more valuable in the context of addressing uncertainties in climate-change projections, especially around climate sensitivity.

**Figure 5.**
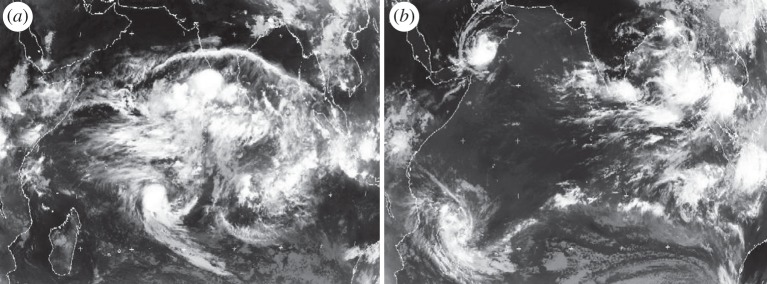
Two infrared satellite images of organized tropical convection over the Indian Ocean for (*a*) 3 May and (*b*) 10 May in 2002. The active phase of the Madden–Julian oscillation is centred over the Indian Ocean on 3 May, with many scales of convective organization embedded within it. A week later on 10 May, the Madden–Julian oscillation has propagated eastwards over Indonesia, leaving two tropical cyclones in its wake and an almost clear Indian Ocean. Weather and climate models still have difficulty in capturing the Madden–Julian oscillation and the richness of its structure.

The understanding of the atmosphere as a multi-scale system and of the role of scale interactions in determining weather and climate variability has developed strongly, especially following the major TOGA COARE^1^ experiment in 1992–1993 [8]. As a result, it has become increasingly clear that the inherent assumption in traditional parametrization schemes that there is no coupling between dynamics and physics on the unresolved scales is now being challenged. This is particularly true for cumulus convection where upscale energy cascades are now recognized as a fundamental part of organized convection, especially in the tropics (e.g. mesoscale squall lines, tropical cyclones and Madden–Julian oscillations; figure 5). This has led to the development of stochastic approaches to physical parametrizations, which attempt to represent unresolved processes and their effects on the dynamics at the resolved scale [9,10]. What this essentially says is that models, because of their coarse resolution, miss some of the nonlinear processes that are fundamental to the atmosphere as a chaotic system.

These stochastic approaches are both physically appealing and also proving very effective in increasing ensemble spread and reducing systematic model biases. As a specific example, stochastic kinetic energy backscatter schemes are designed to reduce the excessive dissipation at small scales by scattering a fraction of the dissipated energy upscale where it acts as a forcing for the resolved-scale flow. The forcing pattern in the scheme is used to describe the spatial–temporal correlations of the backscattered energy, and as Berner *et al.* [10] discussed, the best results are obtained for flow-dependent formulations of the unresolved processes, such as mesoscale convective disturbances. It is now the case that the ensemble spread matches the ensemble mean error, so that the ensemble system is no longer under-dispersive.

With the availability of substantially enhanced computing power, ultra-high-resolution process studies of multi-scale systems, such as tropical organized convection, are now increasingly used to provide the spectral characteristics of the upscale energy cascade and to aid the development of improved stochastic physics parametrizations [11]. The potential for such studies to provide a major breakthrough in the development of stochastically based parametrizations is considerable [12].

Finally, much progress has been made in ensemble systems for numerical weather prediction so that the uncertainties from the initial conditions and from model formulation are both represented. Probabilistic methods are now fundamental to weather forecasting on all scales, including now-casting at the cloud system-resolving scale of 1–2 km. Further progress is likely to be achieved through ongoing reductions in model biases from improved parametrizations and through more innovative approaches to defining initial condition uncertainties, using ensemble data assimilation [13]. Both factors will lead to increased forecast skill and more reliable estimates of probabilities, especially related to extreme events.

## 3. ‘Predictability in the midst of chaos’: why seasonal and decadal forecasting is possible?

In 1981, Charney & Shukla [14] wrote ‘It is shown by numerical simulation that the variability of average pressure and rainfall for July due to short-period flow instabilities occurring in the absence of boundary anomalies can account for most of the observed variability at mid-latitudes but not at low latitudes. On the basis of the available evidence it is suggested that a large part of the low-latitude variability is due to boundary anomalies in such quantities as sea surface temperature, albedo and soil moisture, which, having longer time constants, are more predictable than the flow instabilities'. And so the concept of seasonal prediction was born.

In terms of the Lorenz model, the concept of extended range predictability associated with an external forcing, but still within a chaotic system, was considered by Palmer [15]. He showed that if a forcing, *f*, is included in the Lorenz equations (3.1), then the residence time in the regimes of the Lorenz attractor can change in a predictable way; in other words, the climatic response is predictable, even when the forcing is weak,
3.1
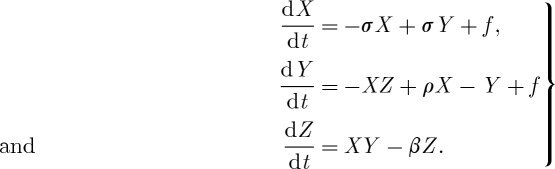

**Figure 6.**
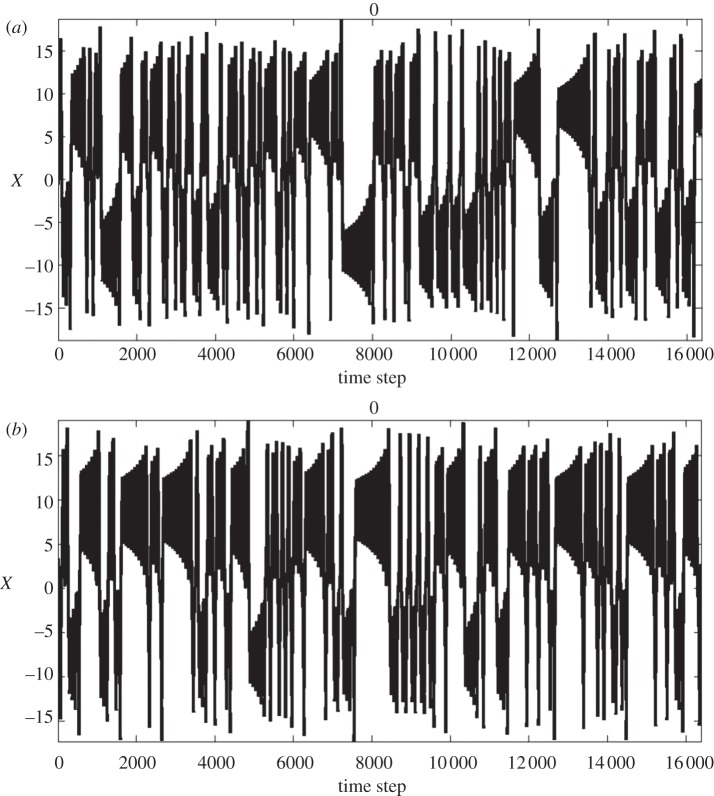
Examples of the time series of the *X* variable in the Lorenz model, evolving on the Lorenz attractor for (*a*) no external forcing and for (*b*) strong external forcing. Changes in probability of the upper regime/lower regime are affected predictably by the imposed ‘forcing’.

Using the Lorenz model and varying the strength of the forcing, it can also be shown that the number and spatial patterns of regimes remain the same, but their frequency of occurrence is changed (figure 6). This is essentially what Charney & Shukla [14] had surmised, at least for the tropical atmosphere, where El Nino, in particular, affects regimes of tropical weather, and indeed mid-latitude weather, especially over the western USA. On the other hand, it is possible that for larger forcings, the number and pattern of regimes can change. An important question is whether anthropogenic climate change due to increasing greenhouse gases constitutes a strong enough forcing to lead to a population of new regimes.

In terms of seasonal to decadal prediction, the predictability of the system resides primarily in the oceans, where the greater thermal capacity and the much longer dynamical time scales for adjustment impart a memory to the coupled ocean–atmosphere system, which exceeds that for the atmosphere alone by several orders of magnitude. Nevertheless, the ocean, like the atmosphere, is a chaotic, nonlinear system, and so an ensemble approach to seasonal to decadal prediction is fundamental to forecasting on these time scales also.

**Figure 7.**
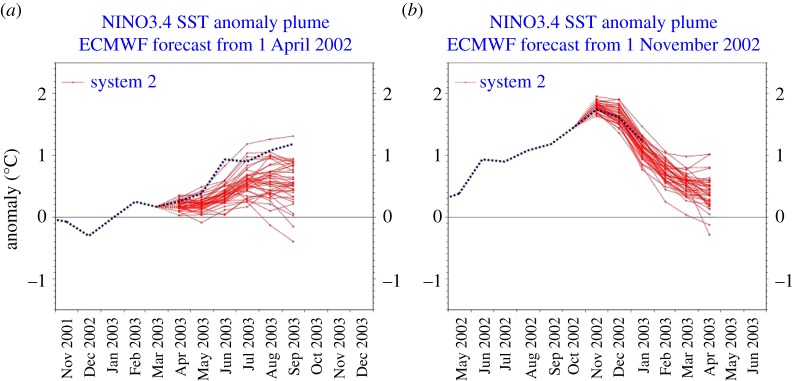
Two contrasting ensemble seasonal forecasts from the European Centre for Medium-range Weather Forecasts (ECMWF) for the evolution of El Nino. (*a*) The initiation of El Nino is difficult to forecast owing to stochastic forcing from the atmosphere, e.g. westerly wind events. (*b*) Decay of an El Nino is more predictable owing to the role of equatorial ocean dynamics.

The latest developments in seasonal to decadal forecasting involve fully coupled models of the ocean and atmosphere, both of which have to be initialized for the current state of the climate system from observations. As in weather forecasting, the ensemble prediction systems typically consider initial condition uncertainty and model uncertainty, using stochastic physics. And as in weather forecasting, the predictability, at any point on the globe and for certain states of the coupled system, is itself predictable. For example, El Nino has predictable effects around the global tropics and over North and South America, and certain phases of El Nino are more predictable than others (figure 7).

**Figure 8.**
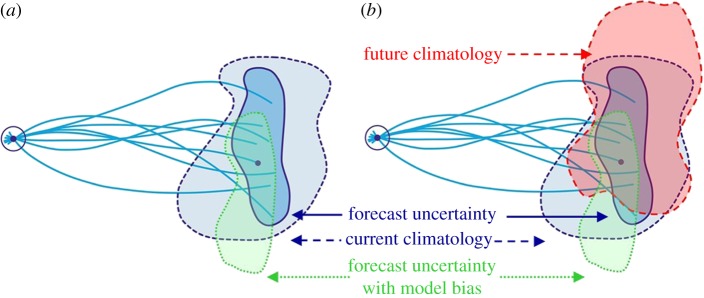
Schematic of ensemble prediction system on seasonal to decadal time scales based on figure 1, showing (*a*) the impact of model biases and (*b*) a changing climate. The uncertainty in the model forecasts arises from both initial condition uncertainty and model uncertainty.

Unlike weather forecasting, however, model-specific biases grow more strongly in a fully coupled system, to the extent that the distribution of probable outcomes in seasonal to decadal forecasts may not reflect the observed distribution (figure 8*a*), and thus the forecasts may not be reliable. It is essential, therefore, that forecast reliability is assessed using large sets of model hindcasts.^2^ This enables the forecast probabilities to be calibrated based on past performance and the model bias to be corrected. However, these empirical correction methods are essentially linear and yet we know that the real system is highly nonlinear. As Turner *et al.* [16] have demonstrated, there is inherently much more predictive skill if improvements in model formulation could be made that reduce these biases, rather than correcting them after the fact.

It is also the case that model-specific biases, both in the mean state and in the internal variability, lead to under-dispersion in the ensemble. This has led to the use of multi-model ensembles in which the differing model-specific biases allow the forecast phase space to be sampled more completely with therefore greater reliability in the ensemble prediction system [17]. However, it has to be recognized that, compared with the stochastic parametrization approach, the multi-model ensemble is a rather ‘ad hoc’ concept and, as discussed below, is dependent on those models that happen to be available at the time of forecasts.

The process of forecast calibration using hindcasts presents some serious challenges, however. Firstly, the observational base has improved substantially over the last few decades, especially for the oceans, and so the skill of the forecasts may also improve just because of better-defined initial conditions. Secondly, the process of calibration assumes that the current climate is stationary, but there is clear evidence that the climate is changing (see the Fourth Assessment Report of the Intergovernmental Panel on Climate Change (IPCC)), especially in temperature; even in the UK, the signal is beginning to emerge in the last two decades. This means, for example, that the UK seasonal forecast for the winter of 2009–2010 gave a 20 per cent chance of a cold winter when calibrated against the last 40 years, but a much higher chance (over 40%) if the system had been calibrated against the last 10 years.

Although both the limited nature of the observational base and a changing climate pose some problems for seasonal prediction, for decadal prediction, they are extremely challenging. There is decadal predictability in the climate system through phenomena such as the Atlantic multi-decadal oscillation and the Pacific decadal oscillation [18,19], but our understanding of these phenomena is still limited largely owing to the paucity of ocean observations. However, decadal prediction effectively bridges the gap between seasonal prediction and climate-change projections. It therefore has an important role to play in understanding climate sensitivity, as a significant part of the predictability on decadal time scales comes from changing levels of greenhouse gas concentrations. So while operational seasonal prediction is now well established, it will be some years before decadal prediction can be used with confidence, but the potential is huge [20].

## 4. Uncertainty in climate-change projections

**Figure 9.**
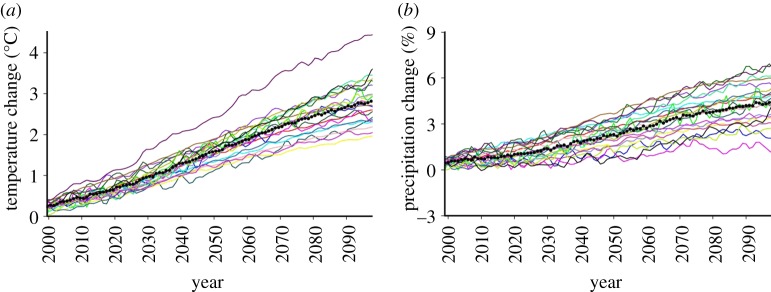
(*a*) Response of the annual mean surface temperature and (*b*) precipitation to Special Report on Emission Scenarios A1B emissions, in 21 climate models that contributed to the IPCC Fourth Assessment Report. The solid black line is the multi-model mean.

Uncertainty in climate-change projections presents some different challenges from uncertainty in weather forecasting or seasonal prediction. The influence of the ocean initial conditions is small beyond a decade or so, and ignoring the uncertainties in the emission scenarios, the major source of uncertainty then comes from the formulation of the models ([21]; figure 9), related particularly to the sensitivity of the climate system to greenhouse gas forcing.

Uncertainty in climate-change projections^3^ has traditionally been assessed using multi-model ensembles of the type shown in figure 9, essentially an ‘ensemble of opportunity’. The strength of this approach is that each model differs substantially in its structural assumptions and each has been extensively tested. The credibility of its projection is derived from evaluation of its simulation of the current climate against a wide range of observations. However, there are also significant limitations to this approach. The ensemble has not been designed to test the range of possible outcomes. Its size is too small (typically 10–20 members) to give robust estimates of the most likely changes and associated uncertainties and therefore it is hard to use in risk assessments.

As already noted, much of the uncertainty in the projections shown in figure 9 comes from the representation of sub-gridscale physical processes in the model, particularly cloud-radiation feedbacks [22]. More recently, the response of the carbon cycle to global warming [23] has been shown to be important, but not universally included yet in the projections. A more comprehensive, systematic and quantitative exploration of the sources of model uncertainty using large perturbed-parameter ensembles has been undertaken by Murphy *et al.* [24] and Stainforth *et al.* [25] to explore the wider range of possible future global climate sensitivities. The concept is to use a single-model framework to systematically perturb poorly constrained model parameters, related to key physical and biogeochemical (carbon cycle) processes, within expert-specified ranges. As in the multi-model approach, there is still the need to test each version of the model against the current climate before allowing it to enter the perturbed parameter ensemble. An obvious disadvantage of this approach is that it does not sample the structural uncertainty in models, such as resolution, grid structures and numerical methods because it relies on using a single-model framework.

As the ensemble sizes in the perturbed ensemble approach run to hundreds or even many thousands of members, the outcome is a probability distribution of climate change rather than an uncertainty range from a limited set of equally possible outcomes, as shown in figure 9. This means that decision-making on adaptation, for example, can now use a risk-based approach based on the probability of a particular outcome.

This concept was extended further by Murphy *et al.* [26] to regional climate change over Europe, based on regional model downscaling from the global model perturbed parameter ensemble. It also included a Bayesian statistical framework designed to support the generation of probabilities constrained by a wide range of observational metrics. This essentially allowed the members of the ensemble to be weighted depending on their ability to reproduce the current climate. The Bayesian framework also accounts for additional model structural uncertainty using the multi-model ensemble.

**Figure 10.**
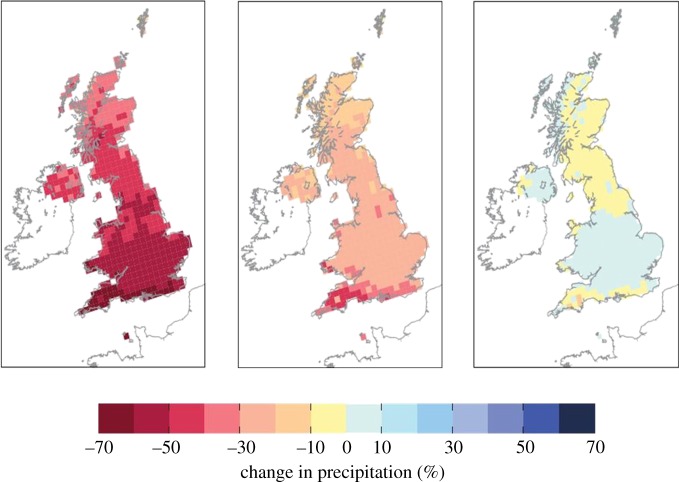
Three potential scenarios of summer rainfall in the 2080s for a high emission scenario (from UKCP09) expressed as a percentage change from the current climate. The middle image shows the central estimate (50% probability) while the left (right) images represent the scenarios where it is unlikely (i.e. 10% probability) that the rainfall may be less (more) than the depicted changes.

This approach was used to produce the 2009 UK Climate Projections (UKCP09), the first example of a fully probabilistic approach to climate-change projection (http://ukclimateprojections.defra.gov.uk/). UKCP09 essentially moved climate-change projection from uncertainty to probability. Future scenarios of climate change can now be given in terms of probabilities of their occurring rather than just being equally likely outcomes of global warming (figure 10). This means that users can assess their vulnerability to different scenarios of climate change and decide what level of risk they are prepared to take based on their own exposure and the probability of that scenario happening. UKCP09 was the first example of a systematic approach to delivering probabilistic information on future regional climate scenarios and it has its limitations. For example, it does not sample uncertainty associated with the resolution of the global driving model, which is now recognized to be important [27] for representing synoptic weather systems with some fidelity.

**Figure 11.**
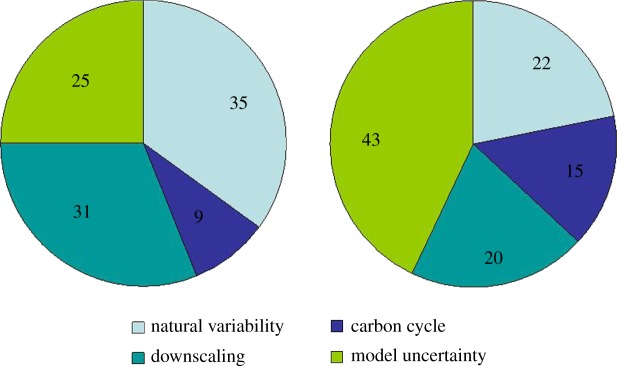
Example of the partitioning of uncertainty in projections of southeast England rainfall for the (*a*) 2020s and (*b*) 2080s from UKCP09.

The other distinct advantage of perturbed parameter ensembles is the ability to quantify the sources of uncertainty and how the sources of uncertainty evolve with the lead time of the forecast. Figure 11 shows an example of where the uncertainties come from in the UKCP09 scenarios for the 2020s and 2080s, in this case, for winter rainfall in southeast England. Of course, as figure 9 indicates, the overall uncertainty increases with time, but the origins of that uncertainty also change. For near-term projections, natural internal variability and regional downscaling dominate the uncertainty, and as suggested by Hawkins & Sutton [21], model uncertainties, including the carbon cycle, dominate at longer lead times.

By delving deeper, it is also possible to identify the particular parameters that contribute the most to the model uncertainty and focus basic research and model development on those science areas. Likewise, the uncertainty from internal variability may be reduced, at least in the near-term projections, through initializing the model with the current state of the climate system. Nevertheless, because the climate is a chaotic system and contains natural variability on all time scales, there is a level of uncertainty that will always exist however much the model uncertainty is reduced.

## 5. Concluding remarks

This paper has considered how Lorenz's theory of the atmosphere (and ocean) as a chaotic, nonlinear system pervades all of weather and climate prediction and how this has influenced the development of probabilistic ensemble prediction systems on all forecast lead times. It has also shown that the sources of uncertainty are not confined to the initial conditions, the basis of the Lorenz model, but that model uncertainty plays a critical role on all time scales.

It is important, however, to distinguish between model uncertainty that arises from imperfect knowledge of the real system, such as the representation of the carbon cycle, and uncertainty that comes from sub-gridscale phenomena that are understood quite well, but are inadequately represented because of the resolution of the model. In weather forecasting, there has been a continuous drive to higher-and-higher resolution with substantial benefits in terms of model performance and forecast skill. Furthermore, recent studies with ultra-high-resolution (approx. 3 km) global models, the so-called cloud system-resolving models, have shown a remarkable ability to capture the multi-scale nature of tropical convection of the type seen in figure 4 [28]. However, the resolution of climate models, still typically 100 km or more, has been constrained fundamentally by a lack of computing resources [29], even though there is compelling evidence to suggest significant improvements in climate model performance with higher horizontal and vertical resolution in both the atmosphere and ocean [27].

Finally, Lorenz's theory of the atmosphere (and ocean) as a chaotic system raises fundamental, but unanswered questions about how much the uncertainties in climate-change projections can be reduced. In 1969, Lorenz [30] wrote: ‘Perhaps we can visualize the day when all of the relevant physical principles will be perfectly known. It may then still not be possible to express these principles as mathematical equations which can be solved by digital computers. We may believe, for example, that the motion of the unsaturated portion of the atmosphere is governed by the Navier–Stokes equations, but to use these equations properly we should have to describe each turbulent eddy—a task far beyond the capacity of the largest computer. We must therefore express the pertinent statistical properties of turbulent eddies as functions of the larger-scale motions. We do not yet know how to do this, nor have we proven that the desired functions exist’. Thirty years later, this problem remains unsolved, and may possibly be unsolvable.

So how much will uncertainties in climate-change predictions of the large-scale reduce if models are run at 20, 2 or even 0.2 km resolution rather than say 100 km resolution? Equally, we may ask whether there is a certain resolution (e.g. 20 km), where it might be feasible to represent small-scale motions using stochastic equations, rather than trying to resolve them? These questions urgently need answering as the pressures grow on the climate science community to estimate, and if possible reduce uncertainties, and provide more reliable and confident predictions of regional climate change, hazardous weather and extremes.

Nevertheless, however much models improve, there will always be an irreducible level of uncertainty—‘flap of the seagull's wings’—because of the chaotic nature of the system. Even the climate we have observed over the past century or so is only one realization of what the real system might produce.

**Figure 12.**
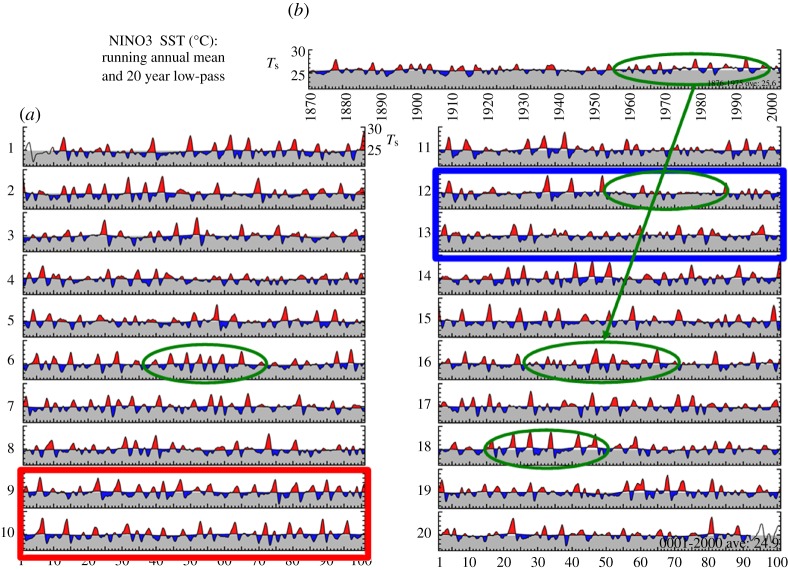
Time series of sea surface temperatures (*T*_s_) for the Nino3 region (5^°^ S–5^°^ N and 150^°^ W–90^°^ W), in the equatorial east Pacific from (*a*) 2000 years of climate model simulation with constant forcing representative of the current climate; (*b*) shows the equivalent time series from observations. Green circles show multi-decadal periods with contrasting El Nino behaviour, including a period in the model's sixteenth century that closely resembles the observed record. Red and blue boxes show extended century-scale periods with contrasting strong and weak El Nino activity, respectively. (Figure courtesy of V. Ramanathan, GFDL, Princeton, NJ, USA).

Figure 12 shows 2000 years of El Nino behaviour simulated by a state-of-the-art climate model forced with present day solar irradiance and greenhouse gas concentrations. The richness of the El Nino behaviour, decade by decade and century by century, testifies to the fundamentally chaotic nature of the system that we are attempting to predict. It challenges the way in which we evaluate models and emphasizes the importance of continuing to focus on observing and understanding processes and phenomena in the climate system. It is also a classic demonstration of the need for ensemble prediction systems on all time scales in order to sample the range of possible outcomes that even the real world could produce. Nothing is certain.

## Footnotes

1 The Tropical Ocean Global Atmosphere Coupled Ocean Atmosphere Response Experiment (TOGA COARE) was a large international field experiment conducted in 1992–1993 to study the atmospheric and oceanic processes over the region of the western Pacific known as the ‘warm pool’.

2 Hindcasts are essentially predictions performed on past cases where the outcome is known. The same system is used for the hindcasts as for the forecasts, with the hindcasts being initialized from the observations at that time. Typical hindcast sets consist of seasonal predictions performed every three months for the last three to four decades.

3 The term ‘projections’ is used here in place of ‘predictions’ because they depend strongly on the emission scenario being used. In addition, the current practice is that these projections are not initialized from the present state of the climate system. In that respect, they do not fit the traditional view of predictability espoused by Lorenz, although natural, internal variability and multi-scale stochastic processes are still important components of the uncertainty.
